# Epilepsia partialis continua complicated by disseminated tuberculosis and hemophagocytic lymphohistiocytosis: a case report

**DOI:** 10.1186/s13256-019-2092-x

**Published:** 2019-06-24

**Authors:** Gashirai K. Mbizvo, Isabel C. Lentell, Clifford Leen, Huw Roddie, Christopher P. Derry, Susan E. Duncan, Kristiina Rannikmäe

**Affiliations:** 1 Muir Maxwell Epilepsy Centre, Centre for Clinical Brain Sciences, The University of Edinburgh, 20 Sylvan Place, Edinburgh, EH9 1UW UK; 2 Department of Clinical Neurosciences, Western General Hospital, Edinburgh, UK; 3 Haematology, Cambridge University Hospitals NHS Foundation Trust, Cambridge, UK; 4 Regional Infectious Diseases Unit, Western General Hospital, Edinburgh, UK; 5 Department of Haematology, Western General Hospital, Edinburgh, UK; 6 Department of Clinical Neurosciences and Sleep Medicine, Centre for Clinical Brain Sciences, The University of Edinburgh, Edinburgh, UK; 7 Forth Valley Royal Hospital, Larbert, and Usher Institute of Population Health Sciences and Informatics, The University of Edinburgh, Edinburgh, UK

**Keywords:** Epilepsia partialis continua, Epilepsy, Seizures, Etoposide, Anticonvulsants, Immunosuppression, Hemophagocytic lymphohistiocytosis, Macrophage activation syndrome, Tuberculosis, Status epilepticus

## Abstract

**Background:**

We describe a patient copresenting with epilepsia partialis continua, tuberculosis, and hemophagocytic lymphohistiocytosis. To our knowledge, this is the first documented case of this triad.

**Case presentation:**

A 54-year-old black South African woman presented to a hospital in Scotland with an acute history of right-sided facial twitching, breathlessness, and several months of episodic night sweats. Clinical examination revealed pyrexia and continuous, stereotyped, right-sided facial contractions. These worsened with speech and continued through sleep. A clinical diagnosis of epilepsia partialis continua was made, and we provide a video of her seizures. Computed tomographic imaging of the chest and serous fluid analyses were consistent with a diagnosis of disseminated *Mycobacterium tuberculosis*. An additional diagnosis of hemophagocytic lymphohistiocytosis was made following the identification of pancytopenia and hyperferritinemia in peripheral blood, with hemophagocytosis evident in bone marrow investigation. We provide images of her hematopathology. The patient was extremely unwell and was hospitalized for 6 months, including two admissions to the intensive care unit for ventilatory support. She was treated successfully with high doses of antiepileptic drugs (benzodiazepines, levetiracetam, and phenytoin) and 12 months of oral antituberculosis therapy, and she underwent chemotherapy with 8 weeks of etoposide and dexamethasone for hemophagocytic lymphohistiocytosis, followed by 12 months of cyclosporine and prednisolone.

**Conclusions:**

This combination of pathologies is unusual, and this case report helps educate clinicians on how such a patient may present and be managed. A lack of evidence surrounding the coexpression of this triad may represent absolute rarity, underdiagnosis, or incomplete case ascertainment due to early death caused by untreated tuberculosis or hemophagocytic lymphohistiocytosis. Further research is needed.

**Electronic supplementary material:**

The online version of this article (https://doi.org/10.1186/s13256-019-2092-x) contains supplementary material, which is available to authorized users.

## Background

Epilepsia partialis continua (EPC) is a rare seizure disorder usually considered a focal status epilepticus, with a prevalence of less than 1 per 1 million population [1]. The diagnosis is often overlooked because of the extended course of the condition, wherein the usual manifestation of epilepsy as paroxysmal events is entirely replaced by a sustained and prolonged repetition of seizure fragments in rapid succession. These symptoms can continue uninterrupted for days to months or even years [2]. This makes EPC extremely disabling for patients. It is important to correctly identify EPC because it can indicate serious underlying pathology [3], including vascular disorders (thromboembolic disease, intracranial bleeds, and vasculitis) in 15–25% of cases, central nervous system (CNS) inflammation or infection (human immunodeficiency virus [HIV], herpes simplex encephalitis, cat scratch disease, Japanese encephalitis, and neurocysticercosis) in 3–45% [2], neoplasms in 5–19%, and metabolic disorders (diabetic ketoacidosis, hepatic encephalopathy, and hyponatremia) in 0–48% [1, 2, 4–9]. In 23% of EPC cases, the cause remains unknown [1, 2, 4–9]. We report an unusual syndrome of EPC complicated by disseminated tuberculosis (TB) and hemophagocytic lymphohistiocytosis (HLH). HLH is a rare hematological disorder involving hyperactivation of macrophages and lymphocytes, usually presenting in infancy. Adult-onset HLH is rarer still and almost universally fatal without treatment, with median survival of only 1.8–2.2 months [10]. To our knowledge, this is the first documentation of a patient presenting with EPC complicated by TB and HLH. It is likely that such a copresentation is underdiagnosed, given the complexities of making each diagnosis. The lessons learned are likely to be relevant to general physicians, who are most likely to encounter such a patient first, as well as to the various medical specialties likely to share the ongoing care of such a patient, including neurology, infectious diseases, and hematology.

## Case presentation

A 54-year-old black South African woman was admitted to a local hospital in Scotland following several months of episodic night sweats, feverishness, and unexplained weight loss. She then developed frequent episodes of right-sided facial and arm “twitching” with preserved consciousness. These episodes progressively became constant and were exacerbated when she tried to talk. They persisted through sleep. She also complained of progressive breathlessness at rest. She recalled being treated for TB over 20 years previously, in the late 1980s, when she had been living in South Africa. Further details of this were unavailable. Her past medical history also included autoimmune neutropenia and mild anemia that had required no intervention. She had no significant family history. She worked in healthcare and did not smoke tobacco or drink alcohol. Although she lived in the United Kingdom, she visited South Africa each year.

Clinical examination revealed a persistent, low-grade pyrexia and a purpuric rash. She was hemodynamically stable, alert, and able to follow commands. She was tachypneic, and on occasion she required inhaled oxygen support to maintain oxygen saturation at 92–94%. She had continuous, semirhythmic contractions in the muscles on the right side of her face. These movements extended from her mouth to her eyebrows (Additional file 1: Video S1). She had no other neurological deficits, and the remainder of her general examination was unremarkable. A summary of various investigations carried out and the differential diagnoses that were considered is shown in Table 1.

**Table 1:** Summary of investigations

Investigation	Results	Differential diagnosis
Peripheral blood investigations	Pancytopenia: Hemoglobin 86 g/L, mean cell volume 86 fL, white cell count 0.1 × 10^9^/L (neutrophil count 0.00 × 10^9^/L, lymphocyte count 0.17 × 10^9^/L), platelets 18 × 10^9^/L, low natural killer cell levels (at 6% detected in an HIV immunology screen) Raised ferritin: 127,985 μg/L Raised C-reactive protein: 123 mg/L Abnormal liver function: Bilirubin 22 μmol/L, alkaline phosphatase 219 U/L, alanine aminotransferase 27 U/L, γ-glutamyl transferase 356 U/L Raised lactate dehydrogenase: 2567 U/L Normal renal function: Sodium 143 mmol/L, potassium 4.3 mmol/L, urea 4.0 mmol/L, creatinine 52 μmol/L Normal clotting: Prothrombin time 14 seconds, activated partial thromboplastin time 29 seconds, fibrinogen 2.4 g/L Negative HIV test Positive antinuclear antibody and positive anti-Ro (> 100.0 U/mL), negative anti-LA/Sm/Scl70/Jo1	Hemophagocytic lymphohistiocytosis Autoimmune disease (for example, systemic lupus erythematosus or Sjögren’s syndrome, which may cause macrophage activation syndrome) Infection/sepsis Aplastic anemia (for example, idiopathic) Marrow infiltration (for example, acute leukemias) Thrombotic thrombocytopenic purpura, hemolytic uremic syndrome
X-ray	Chest x-ray (Fig. 1): Bilateral perihilar opacification	*Pneumocystis* pneumonia Atypical pneumonia Hypersensitivity pneumonitis Pulmonary tuberculosis
Computed tomography	CT of the chest, abdomen, and pelvis with contrast: Abnormal lungs with widespread acinar ground glass opacification, intralobular thickening, and nodular change in a predominantly perihilar distribution. A moderate-sized right pleural effusion was noted. There were no enlarged axillary, supraclavicular, or mediastinal lymph nodes. There was no evidence of pulmonary embolism. Acalculous cholecystitis was noted.	Pulmonary tuberculosis Lymphoma Sarcoidosis
Lumbar puncture	LP opening pressure not recorded; routine CSF was unremarkable with protein of 0.62 g/L, no white cells, and glucose 3.0 mmol/L (paired serum glucose not recorded).	Nondiagnostic routine CSF biochemistry and cell counts
Microbiology	Routine cultures (blood, urine, sputum, CSF): No growth *Pneumocystis jirovecii* PCR negative, *Legionella* antigen and PCR negative, respiratory mycology negative, routine respiratory culture negative Mycobacterial cultures positive (blood, urine, sputum, CSF): *Mycobacterium tuberculosis*	Disseminated *Mycobacterium tuberculosis* *Note*: Acellular CSF in CNS tuberculosis is possible and well-described [11]

Abbreviations: *g* Grams, *L* liter, *HIV* Human immunodeficiency virus, *μg* Microgram, *mg* Milligram, *μmol* Micromole, *U* Units, *mmol* Millimoles, *mL* milliliter, *CT* Computed tomography, *TB* Tuberculosis, *LP* Lumbar puncture, *CSF* Cerebrospinal fluid, *PCR* Polymerase chain reaction, *CNS* Central nervous system


**Additional file 1: Video S1.** Audio-free video of the patient during epilepsia partialis continua (EPC) seizures. This video was taken during the epilepsia partialis continua (EPC) symptoms, with scalp electroencephalogram (EEG) recording electrodes worn. The video shows that the patient had continuous, stereotyped, right-sided, semirhythmic, contractions in the muscles of the face. These extended from the mouth to the eyebrow. The patient was alert and able to communicate throughout the recording. The differential diagnosis includes cortical myoclonus, which is similarly exacerbated by movement but would normally affect the limbs more diffusely [4]. It also includes generalized epileptic myoclonus (for example, in juvenile myoclonic epilepsy), in which an accompanying history of generalized tonic-clonic seizures and generalized epileptiform discharges on EEG would have been expected [38]. Tremor may look similar to EPC but the larger amplitude in EPC usually distinguishes the two types of movements. Tremor is unlikely to continue during sleep. (MP4 778 kb)


**Fig. 1:**
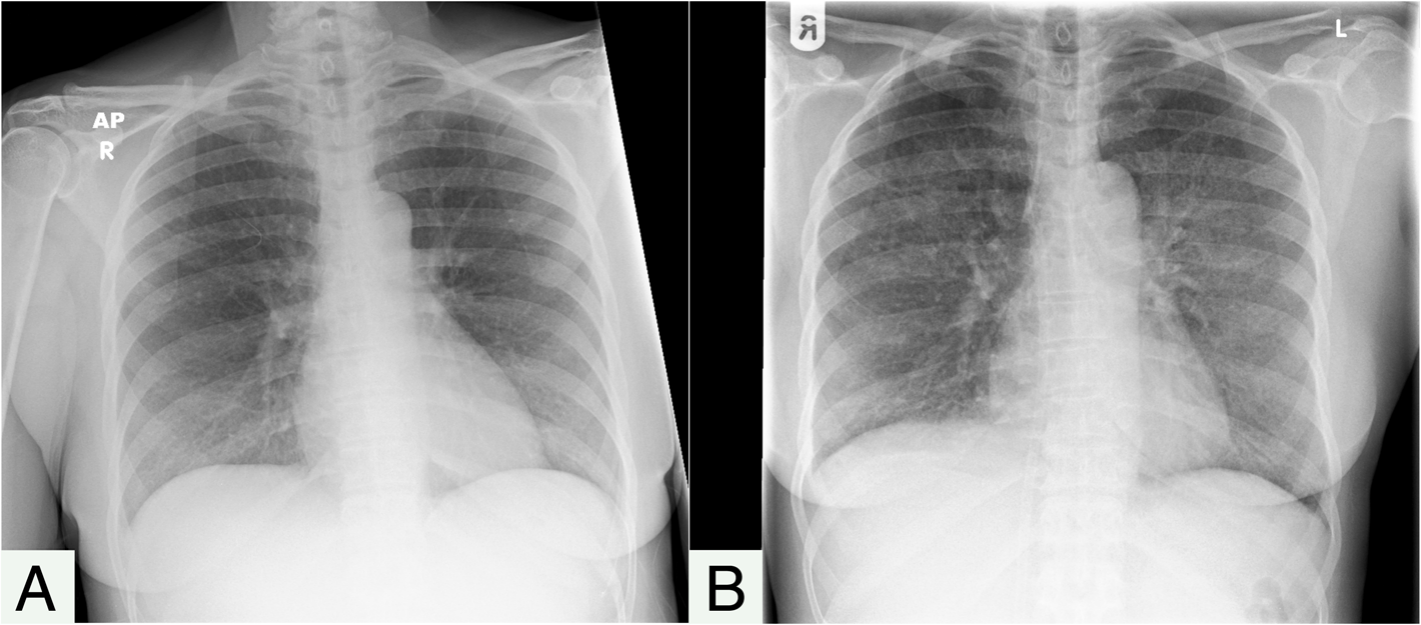
Chest x-ray. **a** Anterior-posterior (AP) film obtained on admission demonstrating normal heart, lung, mediastinum, and bony thorax shadows. **b** Posterior-anterior (PA) film obtained on day 4 of admission to investigate the patient’s acute respiratory failure. The film demonstrates extensive new bilateral acinar opacification in a mainly perihilar distribution throughout all lobes of both lungs in a fairly symmetrical distribution. Normal cardiac contours and pulmonary vascularity are seen

**Fig. 2:**
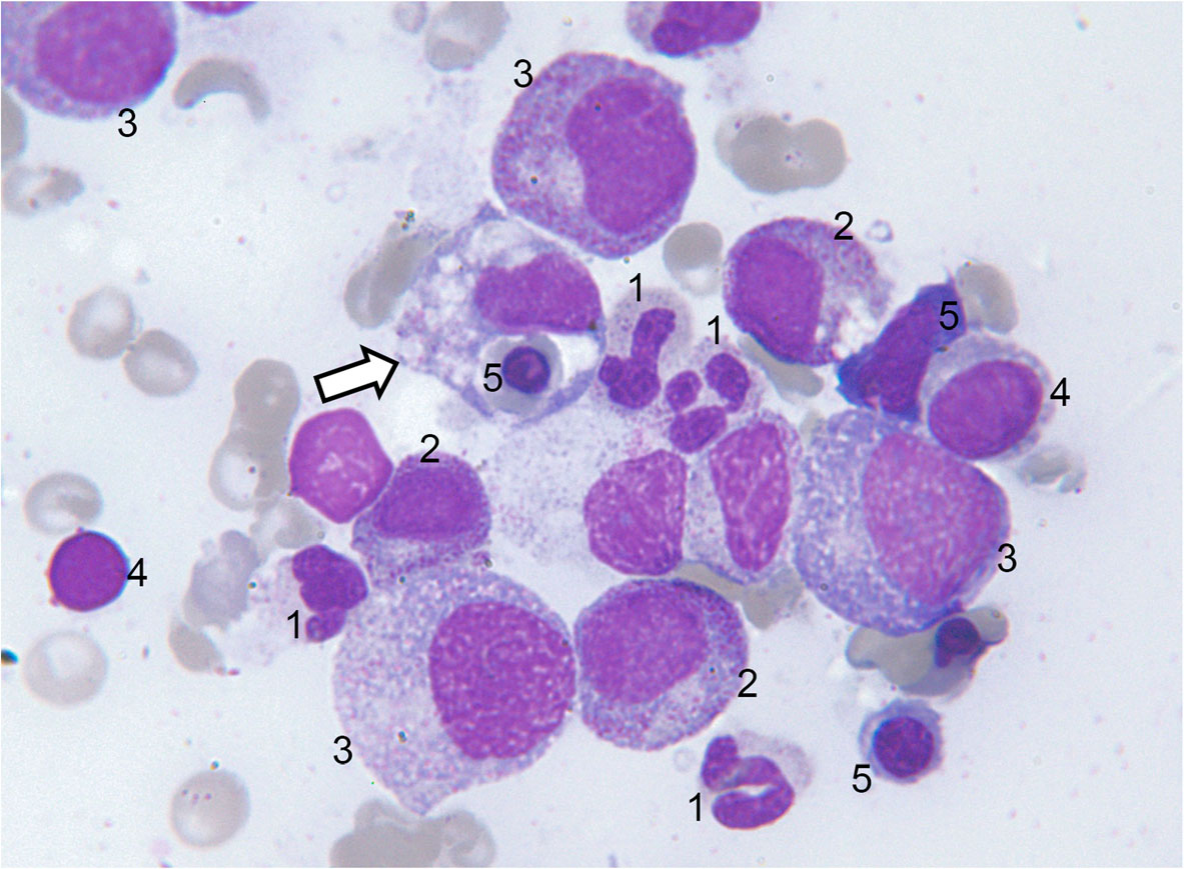
Wright-Giemsa-stained bone marrow aspirate smear. 1 = neutrophil; 2 = myelocyte; 3 = promyelocyte; 4 = lymphocyte; 5 = erythroid precursors at various stages of maturation. The *white arrow* points to an activated macrophage demonstrating hemophagocytosis of an erythroid precursor

**Fig. 3:**
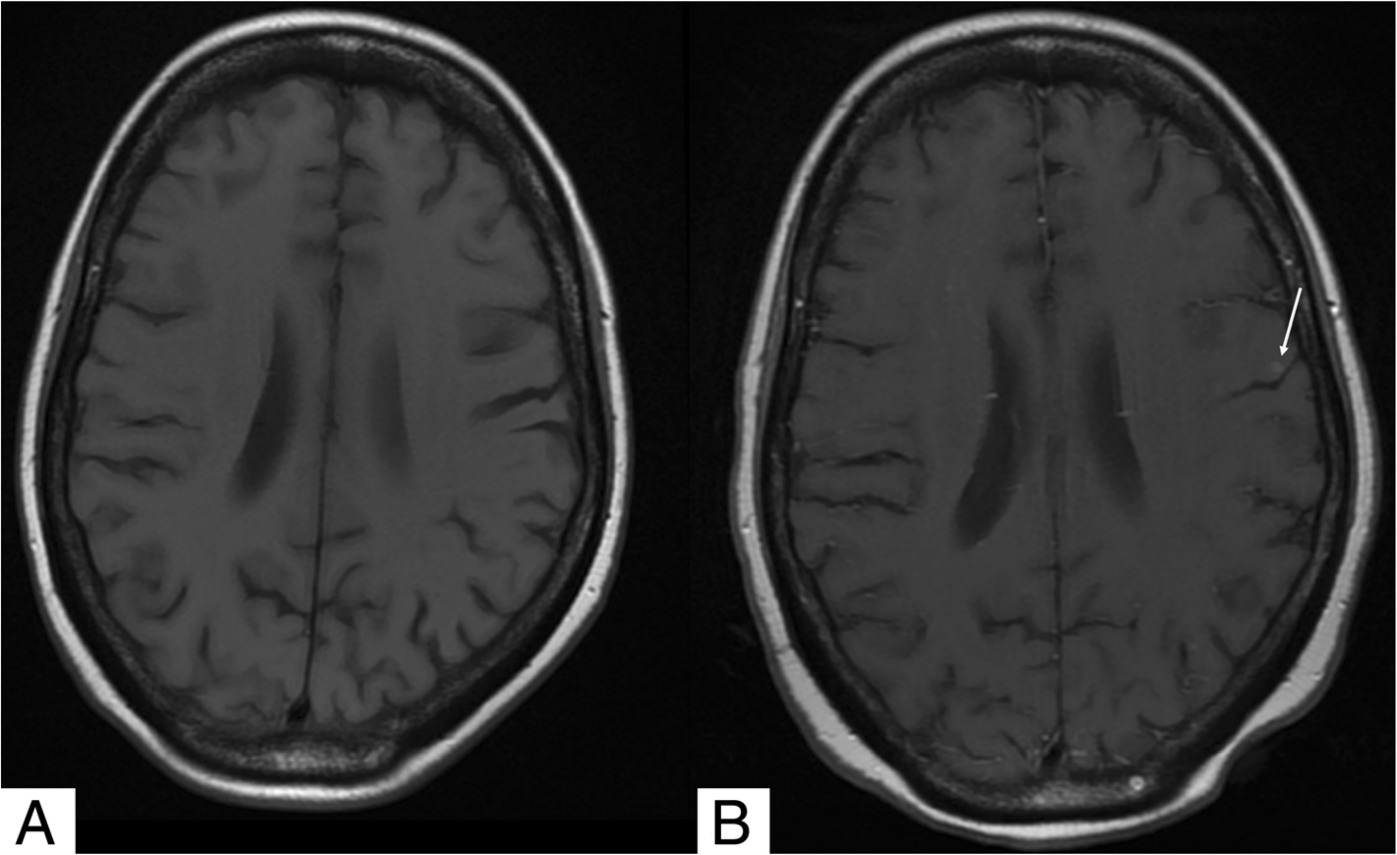
Axial T1 magnetic resonance image of the brain. **a** Precontrast. **b** Postcontrast. A small white focus is seen on the motor strip within the left hemisphere after contrast is added (*white arrow*). The same area is unremarkable before contrast. This contrast-enhancing lesion is a probable tuberculoma. The location of this lesion corresponds to the patient’s right-sided epilepsia partialis continua seizures

**Fig. 4:**
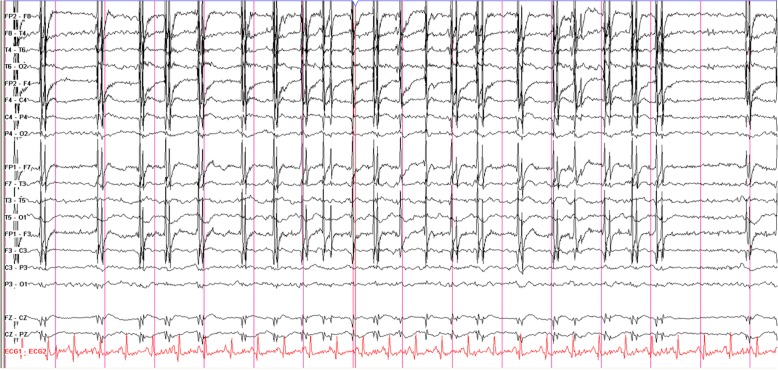
Routine electroencephalogram obtained during the patient’s epilepsia partialis continua symptoms showing muscle artefact

An in-depth timeline of the patient’s inpatient course is provided in Additional file 2: Table S1. She was initially suspected of having meningococcal septicemia, owing to her purpuric rash and pyrexia, and she was commenced on 2 g daily of intravenous (IV) ceftriaxone. Her condition then deteriorated rapidly into acute respiratory failure with new bilateral perihilar opacification evident on chest imaging (Fig. 1), requiring a short period of intensive care for noninvasive ventilation. Ceftriaxone was stopped following negative blood culture results and suspicion that marked thrombocytopenia (lowest platelet count 3 × 10^9^/L) explained her purpura. IV co-trimoxazole 120 mg/kg daily was commenced to treat *Pneumocystis* pneumonia (PCP), and she also received 2 g of meropenem four times daily for 7 days for ongoing pyrexia. She had blood, urine, sputum, and cerebrospinal fluid cultures for mycobacteria. These each isolated *Mycobacterium tuberculosis*. Her pancytopenia and hyperferritinemia prompted bone marrow investigation. A marrow aspirate revealed macrophage activation and hemophagocytosis, supporting a diagnosis of HLH (Fig. 2). A trephine biopsy identified numerous caseating epithelioid granulomata, supporting a diagnosis of TB. Her twitching was investigated with magnetic resonance imaging (MRI) of the brain (with and without contrast). This revealed four small contrast-enhancing foci within the parenchyma of the brain. These were interpreted as likely tuberculomas. One of the lesions was located on the motor strip of her left hemisphere (Fig. 3). This location corresponds somatotopically with her right-sided abnormal facial movements. A clinical diagnosis of EPC was made. Electroencephalography (EEG) revealed muscle artefact but was otherwise normal (Fig. 4). Given the widespread evidence of tuberculous disease, a diagnosis of disseminated TB was made.

**Fig. 5:**
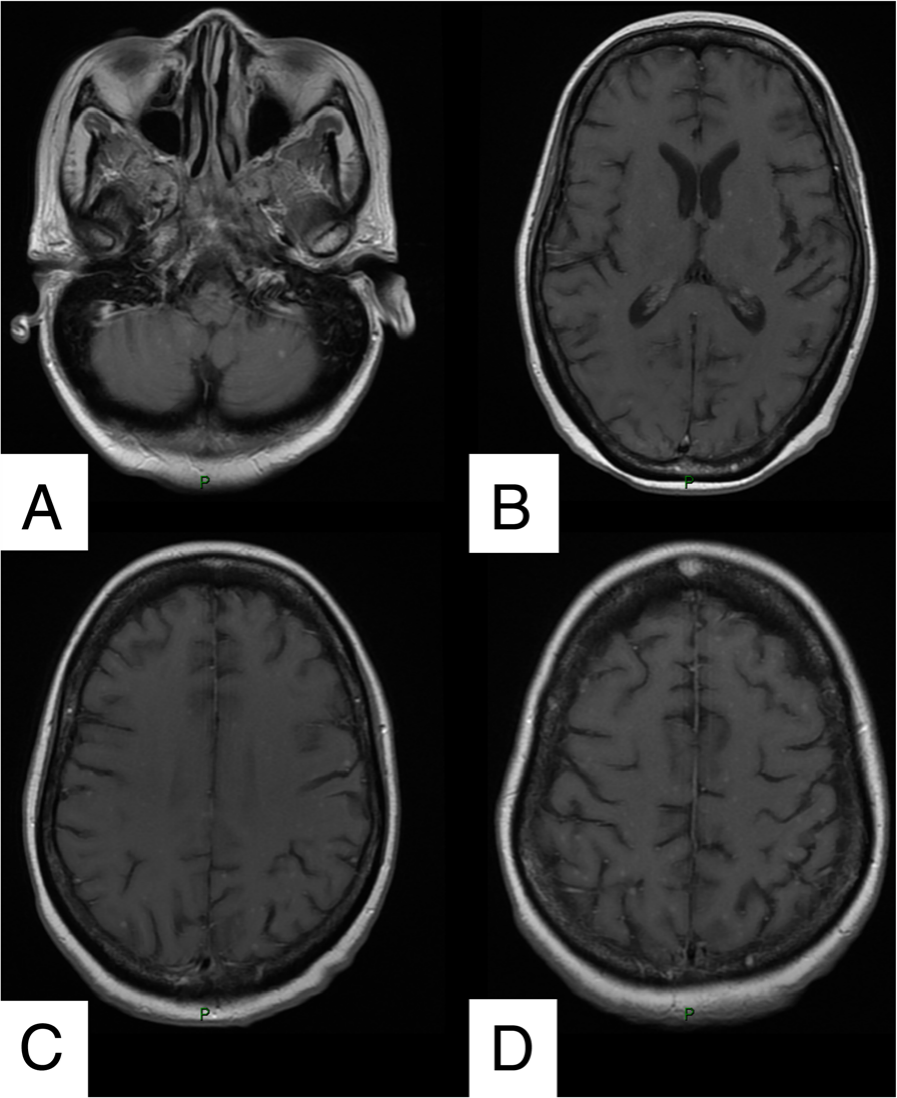
Interval postcontrast axial T1 magnetic resonance images of the brain. These demonstrate many more small white foci (tuberculomata) scattered throughout the brain, particularly in the cerebellar (**a**) and cerebral (**b–d**) parenchyma. Most are 1–2 mm in diameter. A small white focus remains seen on the motor strip within the left hemisphere, corresponding to the patient’s right-sided epilepsia partialis continua seizures

10 mg boluses of IV diazepam were initially given and failed to reduce her continuous focal seizure activity. IV levetiracetam 750 mg daily was then added but demonstrated minimal effect. She was therefore given 10 mg boluses of buccal midazolam and loaded with IV phenytoin at a dose of 17 mg/kg/day. When these were unsuccessful in stopping the seizures, IV levetiracetam was uptitrated to 2500 mg daily, and the IV phenytoin was uptitrated to 550 mg daily. She was also commenced on 1 mg of regular oral clonazepam daily. Her seizures stopped while she was receiving these three medications. She had experienced 7 days of unbroken focal seizure activity in total. A residual right-sided Todd’s paresis was noted, which soon resolved. She required high doses of oral antiepileptic drug (AED) polytherapy to maintain seizure freedom (clobazam 10 mg twice daily, levetiracetam 1500 mg twice daily, and phenytoin 350 mg once daily). Reducing or changing her AEDs proved difficult, with attempts to do so resulting in the reemergence of EPC seizures. Interval MRI of the brain with contrast demonstrated an increased number of enhancing parenchymal foci in keeping with tuberculomata scattered throughout her brain (Fig. 5).

The patient was commenced on a 12-month regimen of oral anti-TB therapy as 6 months of once-daily isoniazid 300 mg, rifampicin 600 mg, pyrazinamide 2 mg, and ethambutol 1000 mg, then 10 months of the isoniazid and rifampicin. Eight weeks of IV etoposide 150 mg/m^2^ were given for HLH (twice weekly for the first 2 weeks, then once weekly for weeks 3–8). Long-term oral steroids were given alongside etoposide as a dexamethasone taper from 18 mg once daily over 8 weeks. Following this, prednisolone was commenced at a 20-mg-daily dose for the duration of anti-TB therapy. Overall, the patient had remained hospitalized for 6 months due to ongoing episodes of unwellness with fevers, respiratory distress (requiring a second brief admission to intensive care, for invasive ventilation), physical mobility problems, and difficulties with memory and speech. During this period, repeated sets of cultures, including blood, urine, sputum, viral throat swabs, blood-borne viruses, and repeat imaging, including of the chest, abdomen, pelvis, and brain, failed to identify a cause of her ongoing pyrexia. A repeat bone marrow aspiration, however, demonstrated ongoing hemophagocytosis, and her ferritin levels remained markedly elevated. Her pyrexia was therefore believed to be a result of ongoing active HLH. She was commenced on long-term oral cyclosporine 250 mg twice daily with a target of normalization of the ferritin levels, which took 12 months. Prednisolone was tapered slowly during this period. The result of her immunologic screening for primary HLH with a granule release assay and perforin expression studies was negative, and she therefore did not undergo further HLH genetic study in light of these results and the increased likelihood that she had HLH secondary to TB. To investigate the possibility of an underlying susceptibility to disseminated TB, the patient had a cytokine profile (including interferon-γ, interleukin 12, and tumor necrosis factor) and GATA2 gene mutation screening, which returned normal results. A biopsy of the lesions in the brain was considered in order to help further characterize the pathological etiology of her seizures, but the risks were believed to outweigh the benefits because she had responded to AEDs and was receiving treatment for both TB and HLH.

Upon discharge, she was seizure-free and experienced a gradual improvement in function, although she remained unable to work and required intensive outpatient functional rehabilitation. She had negative repeat fluid culture results for *M. tuberculosis*, and she was no longer pyrexic or pancytopenic, although her ferritin remained elevated. Her first 12 months of outpatient recovery, during which she completed oral anti-TB treatment, were complicated by five brief readmissions to the hospital with relapses of EPC seizures. Interval MRI scans of the brain during this period demonstrated a gradual reduction and resolution in the number of tuberculomata and progressive atrophy of the left temporal, parietal, and occipital lobes. Her seizure frequency gradually improved over the subsequent 3 years to one or two events per year, and she tolerated stopping phenytoin and remaining on clobazam 10 mg twice daily and levetiracetam 1500 mg twice daily. The patient’s care required frequent collaboration between neurology, infectious disease, and hematology specialists.

## Discussion

Our patient experienced a complex and near-fatal course of EPC, TB, and HLH requiring aggressive and prolonged management. EPC is often a clue to serious underlying pathology, and in our patient, failure to correctly identify EPC could have resulted in the CNS tuberculomas being missed (for example, through use of MRI without contrast only) (*see* Fig. 3). Identification of the tuberculomas guided the decision to lengthen TB eradication to 12 months and to prescribe adjuvant steroids [11]. In Additional file 1, we provide a video demonstrating the patient’s seizures in order to help clinicians recognize the possible semiology of EPC. EPC typically presents with a prolonged period of repetitive muscle jerks to an entire limb, with consciousness preserved. The movements remain uninterrupted throughout the day, worsen with action, and continue during sleep. The symptoms can also be sensory, presenting with continuous somatosensory, proprioceptive, visual, auditory, olfactory, or gustatory symptoms [2, 12]. A diagnosis of EPC should always be made on clinical grounds [6]. EEG is normal in 20% of patients with EPC, and failure of EEG to demonstrate epileptiform activity simply implies that the area of cortical involvement is too small to record or is of a subcortical origin (as seen in myoclonia continua) [1, 5, 6]. The underlying etiology of EPC in our patient was most likely TB with CNS involvement.

The patient’s case was complicated by the co-occurrence of EPC with TB and HLH. HLH is a rare hematological syndrome that is almost universally fatal (owing to multiorgan failure) without treatment [13]. HLH may have been an additional driver, together with TB, of the patient’s repeated respiratory failure episodes [14]. The greatest barrier to prevention of mortality from HLH is delay in diagnosis [15]. It is unlikely that our patient would have survived her respiratory failure without prompt recognition of the HLH and coinitiation of chemotherapy alongside anti-TB therapy. A recent case series of eight patients with TB-HLH reported four cases of respiratory failure requiring invasive ventilation and six deaths despite treatment [14]. HLH is characterized by hemophagocytosis (phagocytosis of erythrocytes, leukocytes, platelets, and their precursors by macrophages) [16, 17]. This is due to dysregulation of activation and proliferation of macrophages in the bone marrow and other tissues. Primary (familial) HLH has a genetic etiology, whereas secondary HLH may be associated with malignancy, autoimmune/rheumatological disease (termed *macrophage activation syndrome* [MAS]) [17, 18], or infection [16, 17]. Although most infection-associated cases can be attributed to the Epstein-Barr virus, TB-associated HLH is also recognized [16]. We propose that in our patient, HLH was secondary to TB, giving rise to a MAS [19–22]. Given her positive anti-Ro antibody, a possible differential diagnosis was Sjögren’s syndrome resulting in MAS, although clinically this was less likely. HLH can present with fever, ataxia, and seizures [23]. Investigations may reveal splenomegaly, cytopenia, hypertriglyceridemia, hemophagocytosis, low/absent natural killer (NK) cell activity, raised ferritin, or elevated interleukin 2 levels [24]. Various genetic mutations may be found, including *PRF1*, *UNC13D*, *STX11*, *STXBP2*, *RAB27*, and *XLP* [25]. Genetic testing is the gold standard for diagnosing primary HLH. However, because the results can take weeks to months, this test is not often helpful in acutely differentiating primary from secondary HLH [26]. Immunological screening investigations, as were done for our patient, greatly facilitate this diagnostic process because they can quickly diagnose and categorize patients with primary HLH. The available immunological tests include NK cell function testing, perforin expression testing, and measurement of CD107a upregulation to evaluate NK cell degranulation [26]. MRI of the brain in HLH may show discrete nodular enhancing parenchymal lesions [27]. It remains possible, therefore, that seizures in our patient were potentiated by HLH, particularly if findings on her MRI could be coattributed to HLH. The only way to resolve this would have been to obtain a lesional biopsy of the brain, which was decided against on clinical grounds and by patient choice.

There have been a small number of studies reporting the co-occurrence of EPC and TB, identifying ten patients (summarized in Additional file 3: Table S2) [5, 9, 28, 29]. Taken together with our patient’s case, the evidence suggests TB can cause EPC through a mechanism of vasculitic infarctions or a mechanism of tuberculoma formation. The reported duration of EPC in TB typically ranges from hours to 10 days. The mainstay of treating EPC is to focus therapy on the underlying condition [1]. In their case report, Bataduwaarachchi *et al.* [28] were able to terminate seizures by eradicating TB using a 2-month induction of oral isoniazid, rifampicin, pyrazinamide, and intramuscular streptomycin, followed by 10 months of isoniazid and rifampicin. Adjunctive steroids were given as IV methylprednisolone pulses for 3 days, followed by a 1-month oral taper. AEDs were 200 mg of sodium valproate given three times daily and 10 mg of clobazam given twice daily. The remaining studies did not specifically report how TB was eradicated or whether this reduced the EPC seizures. All patients required high doses of AEDs. In the context of this limited pool of evidence describing how to specifically manage EPC caused by TB, we have been able to provide anecdotal corroborative evidence that a 12-month anti-TB therapy and steroid regimen [11] can be effective in eradicating TB and reducing seizures in EPC, alongside high doses of AEDs. We await other documented reports of EPC complicated by TB and HLH. Indeed, although there is an association between HLH and epilepsy, EPC-associated HLH is also yet to be reported. Limited evidence surrounding the coexpression of these three diseases may represent absolute rarity, underdiagnosis, or incomplete case ascertainment due to early death of untreated TB or HLH.

There are few prospective trials guiding the treatment of HLH in adults [30], and treatment has been based largely on the HLH-94 study on children aged < 16 years with primary HLH [13, 31]. This highlights the rarity of HLH in adults. Anecdotally, HLH chemotherapy might be avoidable in secondary HLH, when the patient is well, by treating the underlying cause only [15]. This was not the case for our patient, and she required treatment with the HLH-94 protocol (8 weeks of etoposide and dexamethasone followed by cyclosporine and oral steroids). This protocol is normally used as a bridge to allogenic hematopoietic cell transplantation in primary HLH, whereas secondary HLH normally depends on successfully treating the underlying cause [31]. Our patient demonstrated no further evidence of TB, but it took a long time for her hyperferritinemia to settle following TB eradication. In this context, it became difficult to know how long cyclosporine and steroids should have been continued, another area lacking evidence. The more recent HLH-2004 trial used only adjuvant cyclosporine with etoposide and dexamethasone in children with HLH aged < 18 years for a total of 8 weeks [32]. It is possible that the cyclosporine potentiated our patient’s seizures, because these are known side effects of the drug. However, the benefits of treating HLH aggressively in our patient, given how unwell she had been in association with this disease, were believed to outweigh the risks of triggering an ongoing focal epilepsy that had been therapy-responsive, did not generalize, and did not compromise her airway (and therefore was more benign than uncontrolled and potentially fatal HLH). The other drugs that have been tried in HLH are equally or potentially more toxic than cyclosporine and include a combination of cyclophosphamide, vincristine, and prednisone [33]; a combination of cyclophosphamide, doxorubicin, vincristine, and prednisolone (CHOP) [34]; or doxorubicin, etoposide, and methylprednisolone (DEP) [30]. In the context of a scant evidence base, treating HLH and EPC is likely to be complex in all cases, and it will be important for clinicians to talk through the potential risks and benefits carefully with patients in helping them come to a decision on their preferred therapeutic avenue.

From a public health perspective, the case highlights some of the difficulties in ascertaining a complete history of TB exposure, completed therapy, or documented eradication for clinicians seeing patients in whom exposure is historic and within resource-poor settings. Our patient’s recollected experience of TB and TB therapy dated back to more than 20 years prior, during the apartheid era of South Africa, when healthcare provision and documentation for black Africans was very poor [35]. For all patients who are healthcare workers in Scotland, mandatory checks for TB disease/immunity are completed before clinical duties are allowed to commence [36]. They include assessment of personal or family history of TB, tuberculin skin testing (or interferon-γ testing) plus or minus bacillus Calmette–Guérin vaccination where required, and routine chest x-ray for those from high-risk countries, plus or minus TB treatment if indicated. Further details are provided in the Scottish Government document “Health Clearance for Tuberculosis, Hepatitis B, Hepatitis C and HIV for New Healthcare Workers with Direct Clinical Contact with Patient” [36]. This policy applies to all NHS Scotland healthcare workers having direct clinical contact with patients, including new or returning healthcare workers, and healthcare workers changing to a new NHS employer (unless providing valid evidence of screening from their previous NHS employer). The policy was introduced in 2008. In our patient’s specific case, she began working for NHS Scotland prior to this date and was not screened for TB. She did have a baseline chest x-ray that demonstrated nothing to suggest past infection with TB (Fig. 1). However, it remains unclear if her TB manifested as a new infection or as reactivation of latent TB. If the case were the latter, it would suggest a potential for value in prospectively screening healthcare workers employed in Scotland prior to 2008 if they have not already been screened through existing mechanisms. Overall, there remains a significantly increased risk of both latent TB and active TB in healthcare workers worldwide compared with the general population [37].

## Conclusions

We describe the complex interdisciplinary case of a patient presenting with episodic feverishness, continuous facial twitching, and pancytopenia. She has been diagnosed with EPC complicated by TB and HLH. These are difficult diagnoses to make, and we provide clinicians with a helpful video of the patient’s EPC seizures and annotated images of the hemophagocytosis viewed on marrow aspirate. The case educates clinicians to persist with investigating for an underlying cause when presented with a patient with EPC (including the importance of using contrast on the MRI brain scan if an unenhanced scan is unremarkable). It also educates clinicians not to forget to investigate for HLH when faced with a patient with TB presenting with pancytopenia, and it is a reminder that HLH, although usually a pediatric condition, can occur in adults. Although HLH is rare, its very high mortality makes this an important diagnosis not to miss. Clinicians faced with these patients may wish to try a prolonged oral TB eradication schedule (12 months), alongside HLH-specific chemotherapy with 8 weeks of etoposide and dexamethasone. If there remains evidence of HLH disease activity (we used ferritin as the marker), our patient’s case suggests that a maintenance combination of cyclosporine and prednisolone may be helpful. This case also suggests that escalating doses of parenteral benzodiazepines, levetiracetam, and phenytoin initially, followed by continuing high doses of these AEDs orally, may be helpful in managing EPC in a patient with TB and HLH. This is a rare combination of disease, and this report encourages clinicians to remain vigilant about this syndrome, and it should help stimulate further research on the most effective way to manage these patients. The management avenues we tried were helpful anecdotally but would require validation as part of a controlled interventional trial on patients with TB, EPC, and HLH, which is yet to be done.

## Additional files

Additional file 2: **Table S1.** Clinical details and timeline of the patient’s inpatient journey. A timeline detailing the patient’s inpatient journey, including clinical features, investigations, and management. (DOCX 23 kb)

Additional file 3: **Table S2.** Summary of published studies reporting the co-occurrence of epilepsia partialis continua (EPC) and tuberculosis (TB). A case series summarized from the published studies reporting the co-occurrence of epilepsia partialis continua (EPC) and tuberculosis (TB). (DOCX 21 kb)

## Abbreviations

AED: Antiepileptic drug
CNS: Central nervous system
CSF: Cerebrospinal fluid
CT: Computed tomography
EEG: Electroencephalography
EPC: Epilepsia partialis continua
HIV: Human immunodeficiency virus
HLH: Hemophagocytic lymphohistiocytosis
IV: Intravenous
MAS: Macrophage activation syndrome
MRI: Magnetic resonance imaging
NK: Natural killer
TB: Tuberculosis

## References

[1] Cockerell OC, Rothwell J, Thompson PD, Marsden CD, Shorvon SD. Clinical and physiological features of epilepsia partialis continua: cases ascertained in the UK. Brain. 1996;119(Pt 2):393–407. 10.1093/brain/119.2.393 8800935

[2] Mameniskiene R, Wolf P. Epilepsia partialis continua: a review. Seizure. 2017;44:74–80. 10.1016/j.seizure.2016.10.010 28029552

[3] Patel P, Amin N, Patel SB, Morgan C. A fitting tribute to epilepsia partialis continua. Ann Med Surg. 2015;4(3):283–6. 10.1016/j.amsu.2015.08.004 PMC4556781 26468371

[4] Bien CG, Elger CE. Epilepsia partialis continua: semiology and differential diagnoses. Epileptic Disord. 2008;10(1):3–7. 10.1684/epd.2008.0161 18367424

[5] Pandian JD, Thomas SV, Santoshkumar B, Radhakrishnan K, Sarma PS, Joseph S, Kesavadas C. Epilepsia partialis continua—a clinical and electroencephalography study. Seizure. 2002;11(7):437–41. 10.1053/seiz.2001.0646 12237069

[6] Thomas JE, Reagan TJ, Klass DW. Epilepsia partialis continua: a review of 32 cases. Arch Neurol. 1977;34(5):266–75. 10.1001/archneur.1977.00500170020003 404996

[7] Mameniskiene R, Bast T, Bentes C, Canevini MP, Dimova P, Granata T, Hogenhaven H, Jakubi BJ, Marusic P, Melikyan G, *et al.* Clinical course and variability of non-Rasmussen, nonstroke motor and sensory epilepsia partialis continua: a European survey and analysis of 65 cases. Epilepsia. 2011;52(6):1168–76. 10.1111/j.1528-1167.2010.02974.x 21320117

[8] Phabphal K, Limapichat K, Sathirapanya P, Setthawatcharawanich S, Geater A. Clinical characteristics, etiology and long-term outcome of epilepsia partialis continua in adult patients in Thailand. Epilepsy Res. 2012;100(1–2):179–87. 10.1016/j.eplepsyres.2012.02.009 22386873

[9] Sinha S, Satishchandra P. Epilepsia partialis continua over last 14 years: experience from a tertiary care center from south India. Epilepsy Res. 2007;74(1):55–9. 10.1016/j.eplepsyres.2006.12.003 17292588

[10] Shah AR, Muzzafar T, Assi R, Schellingerhout D, Estrov Z, Tamamyan G, Kantarjian H, Daver N. Hemophagocytic lymphohistiocytosis in adults: an under recognized entity. BBA Clin. 2016;7:36–40. 10.1016/j.bbacli.2016.12.002 PMC5219626 28070498

[11] Thwaites G, Fisher M, Hemingway C, Scott G, Solomon T, Innes J, British Infection S. British Infection Society guidelines for the diagnosis and treatment of tuberculosis of the central nervous system in adults and children. J Infect. 2009;59(3):167–87. 10.1016/j.jinf.2009.06.011 19643501

[12] Bancaud J, Bonis A, Trottier S, Talairach J, Dulac O. Continuous partial epilepsy: syndrome and disease [in French]. Rev Neurol (Paris). 1982;138(11):803–14. 6820177

[13] Schram AM, Berliner N. How I treat hemophagocytic lymphohistiocytosis in the adult patient. Blood. 2015;125(19):2908–14. 10.1182/blood-2015-01-551622 25758828

[14] Zhang Y, Liang G, Qin H, Li Y, Zeng X. Tuberculosis-associated hemophagocytic lymphohistiocytosis with initial presentation of fever of unknown origin in a general hospital: an analysis of 8 clinical cases. Medicine (Baltimore). 2017;96(16):e6575. 10.1097/MD.0000000000006575 PMC5406066 28422850

[15] McClain KL. In: Newberger P, editor. Treatment and prognosis of hemophagocytic lymphohistiocytosis. Waltham; 2018. UpToDate.

[16] Brastianos PK, Swanson JW, Torbenson M, Sperati J, Karakousis PC. Tuberculosis-associated haemophagocytic syndrome. Lancet Infect Dis. 2006;6(7):447–54. 10.1016/S1473-3099(06)70524-2 16790385

[17] Padhi S, Ravichandran K, Sahoo J, Varghese RG, Basheer A. Hemophagocytic lymphohistiocytosis: an unusual complication in disseminated *Mycobacterium tuberculosis*. Lung India. 2015;32(6):593–601. 10.4103/0970-2113.168100 PMC4663863 26664166

[18] Hashmi HRT, Mishra R, Niazi M, Venkatram S, Diaz-Fuentes G. An unusual triad of hemophagocytic syndrome, lymphoma and tuberculosis in a non-HIV patient. Am J Case Rep. 2017;18:739–45. 10.12659/AJCR.903990 PMC5507798 28669977

[19] Le Hô H, Barbarot N, Desrues B. Pancytopenia in disseminated tuberculosis: think of macrophage activation syndrome [in French]. Rev Mal Respir. 2010;27(3):257–60. 10.1016/j.rmr.2010.02.005 20359619

[20] Thiam K, Sagne J, Ndiaye EHM, Cisse MF, Mbaye FBR, Toure NO, Dia Kane Y, Diatta A, Niang S, Kombila UD, *et al.* Hepatic cytolysis and bicytopenia after 15 days of four-drug tuberculosis treatment in Senegal. Med Sante Trop. 2017;27(3):233–4. 10.1684/mst.2017.0714 28947398

[21] Matsuura-Otsuki Y, Hanafusa T, Igawa K, Sato H, Nishizawa A, Yokozeki H. Macrophage activation syndrome triggered by disseminated tuberculosis with tuberculous gumma in a patient with adult-onset Still’s disease and Good’s syndrome. Eur J Dermatol. 2016;26(3):309–11. 10.1684/ejd.2016.2745 27018416

[22] Andre V, Liddell C, Guimard T, Tanguy G, Cormier G. Macrophage activation syndrome revealing disseminated tuberculosis in a patient on infliximab. Joint Bone Spine. 2013;80(1):109–10. 10.1016/j.jbspin.2012.06.022 23286892

[23] Sulaiman RA, Shaheen MY, Al-Zaidan H, Al-Hassnan Z, Al-Sayed M, Rahbeeni Z, Bakshi NA, Kaya N, Aldosary M, Al-Owain M. Hemophagocytic lymphohistiocytosis: a rare cause of recurrent encephalopathy. Intractable Rare Dis Res. 2016;5(3):227–30. 10.5582/irdr.2016.01018 PMC4995419 27672548

[24] George MR. Hemophagocytic lymphohistiocytosis: review of etiologies and management. J Blood Med. 2014;5:69–86. 10.2147/JBM.S46255 PMC4062561 24966707

[25] Zhang K, Filipovich AH, Johnson J, Marsh RA, Villanueva J. Hemophagocytic lymphohistiocytosis, familial. In: Adam MP, Ardinger HH, Pagon RA, Wallace SE, LJH B, Stephens K, Amemiya A, editors. GeneReviews [Internet]. Seattle: University of Washington, Seattle; 2006. [updated 17 Jan 2013].

[26] Rubin TS, Zhang KJ, Gifford C, Lane A, Choo S, Bleesing JJ, Marsh RA. Perforin and CD107a testing is superior to NK cell function testing for screening patients for genetic HLH. Blood. 2017;129(22):2993–9. 10.1182/blood-2016-12-753830 PMC5766842 28270454

[27] Goo HW, Weon YC. A spectrum of neuroradiological findings in children with haemophagocytic lymphohistiocytosis. Pediatr Radiol. 2007;37(11):1110–7. 10.1007/s00247-007-0569-z 17846757

[28] Bataduwaarachchi VR, Tissera N. Seizures in an immunocompromised adolescent: a case report. J Med Case Rep. 2015;9:184. 10.1186/s13256-015-0660-2 PMC4552135 26315723

[29] Kravljanac R, Jovic N, Djuric M, Pekmezovic T. Immune-mediated and inflammatory disease in etiology of epilepsia partialis continua in children [abstract 007]. Epilepsia. 2010;51(Suppl 4):3.

[30] Wang YN, Huang WQ, Hu LD, Cen XA, Li LH, Wang JJ, Shen JL, Wei N, Wang Z. Multicenter study of combination DEP regimen as a salvage therapy for adult refractory hemophagocytic lymphohistiocytosis. Blood. 2015;126(19):2186–92. 10.1182/blood-2015-05-644914 PMC4635114 26289641

[31] Henter JI, Samuelsson-Horne A, Arico M, Egeler RM, Elinder G, Filipovich AH, Gadner H, Imashuku S, Komp D, Ladisch S, *et al.* Treatment of hemophagocytic lymphohistiocytosis with HLH-94 immunochemotherapy and bone marrow transplantation. Blood. 2002;100(7):2367–73. 10.1182/blood-2002-01-0172 12239144

[32] Bergsten E, Horne A, Arico M, Astigarraga I, Egeler RM, Filipovich AH, Ishii E, Janka G, Ladisch S, Lehmberg K, *et al.* Confirmed efficacy of etoposide and dexamethasone in HLH treatment: long-term results of the cooperative HLH-2004 study. Blood. 2017;130(25):2728–38. 10.1182/blood-2017-06-788349 PMC5785801 28935695

[33] Hu Y, Xu J, Wang L, Li J, Qiu H, Zhang S. Treatment of hemophagocytic lymphohistiocytosis with cyclophosphamide, vincristine, and prednisone. Swiss Med Wkly. 2012;142:w13512. 10.4414/smw.2012.13512 22307778

[34] Shin HJ, Chung JS, Lee JJ, Sohn SK, Choi YJ, Kim YK, Yang DH, Kim HJ, Kim JG, Joo YD, *et al.* Treatment outcomes with CHOP chemotherapy in adult patients with hemophagocytic lymphohistiocytosis. J Korean Med Sci. 2008;23(3):439–44. 10.3346/jkms.2008.23.3.439 PMC2526520 18583880

[35] Andersson N, Marks S. Apartheid and health in the 1980s. Soc Sci Med. 1988;27(7):667–81. 10.1016/0277-9536(87)90327-3 3067361

[36] Scottish Government. Health clearance for tuberculosis, hepatitis B, hepatitis C and HIV for new healthcare workers with direct clinical contact with patient. Edinburgh: Author; 2008.

[37] Uden L, Barber E, Ford N, Cooke GS. Risk of tuberculosis infection and disease for health care workers: an updated meta-analysis. Open Forum Infect Dis. 2017;4(3):ofx137. 10.1093/ofid/ofx137 PMC5575844 28875155

[38] Kojovic M, Cordivari C, Bhatia K. Myoclonic disorders: a practical approach for diagnosis and treatment. Ther Adv Neurol Disord. 2011;4(1):47–62. 10.1177/1756285610395653 PMC3036960 21339907

