# Supplementary material for: Epilepsia partialis continua complicated by disseminated tuberculosis and hemophagocytic lymphohistiocytosis: a case report
Source: J Med Case Rep. 2019 Jun 24;13:191. doi: 10.1186/s13256-019-2092-x (PMC6589876; doi:10.1186/s13256-019-2092-x)
Supplement: Supplementary file 2 — Table S1. Clinical details and timeline of the patient’s inpatient journey. A timeline detailing the patient’s inpatient journey, including clinical features, investigations, and management. (DOCX 23 kb) [file 13256_2019_2092_MOESM2_ESM.docx]

**Table S1: Clinical details and timeline of the patient's inpatient journey**

| Time | Clinical summary | Clinical details |
| --- | --- | --- |
| Day 0 | Admission and empirical treatment for infection | - Admitted under general medicine/hematology with **fevers, night sweats, weight loss,** - Purpuric rash noted - **Pancytopenia** with Hb 110 g/L, WCC 3.5 x 10^9 /L, PLT 18 x 10^9/L, **hyperferritinaemia** 4068 μg/L, **raised CRP** 123 mg/L, normal renal function - CXR unremarkable - Initial therapy with ceftriaxone for suspected meningococcal disease |
| Day 1 | Worsening pancytopenia | - Hb 94 g/L, WCC 3.9 x 10^9 /L, **PLT 3** x 10^9/L |
| Day 2 | HLH detected, steroids commenced | - Multiple sets of blood cultures: negative, purpura felt to be related to thrombocytopenia - HIV negative - **Bone marrow aspirate demonstrated hemophagocytosis**, patient **commenced on dexamethasone** initially for this |
| Day 4 | Respiratory failure and admission to ITU, PCP suspected | - **Acute respiratory failure** - Transferred to ITU and commenced CPAP - **CXR showed extensive new bilateral perihilar opacification**, PCP suspected. Similar changes shown on CT-CAP - Ceftriaxone changed to IV co-trimoxazole, also covered with IV tazocin and clarithromycin initially then changed to IV meropenem for 7 days given ongoing temperature spikes |
| Day 6 | ITU stepdown | - **Improved respiratory function**, stepped down to infectious diseases unit on 50% inhaled oxygen |
| Days 9–10 | TB suspected | - **Sputum culture: *Mycobacterium tuberculosis*** - **Bone marrow trephine** biopsy: no micro-organisms but **caseating granulomatous inflammation** seen and suspicious for mycobacterial infection - ***Pneumocystis jirovecii* PCR negative**, *Legionella* antigen and PCR negative, respiratory mycology negative, routine respiratory culture negative - Co-trimoxazole stopped |
| Days 13–24 | Clinical improvement | - **Patient improving clinically**, apyrexial, CXR cleared, no longer requiring oxygen, CRP normalized to 4 mg/L, meropenem stopped - PLT remained low at 48 x 10^9/L, Hb 128 g/L, WCC 4.5 x 10^9 /L - Investigation ongoing |
| Days 25–26 | Further evidence of TB | - **Blood culture: *Mycobacterium tuberculosis*** - **Urine culture: *Mycobacterium tuberculosis*** |
| Days 32–39 | Clinical deterioration, readmitted to ITU, ventilated, anti-TB therapy commenced, chemotherapy added in | - **Acute respiratory failure, re-admitted to ITU, intubated and ventilated** - Required vasopressors, inotropic support, and renal replacement therapy - **Commenced isoniazid, rifampicin, pyrazinamide, ethambutol** - **Worsening pancytopenia** ranging **Hb 86–91** g/L, **platelets 18–25** x 10^9/L, **WCC 0.1–0.3** x 10^9 /L, **neutrophils 0.00–0.20** x 10^9 /L, worsening **hyperferritinaemia ranging 58,999–127,985** μg/L, **etoposide commenced** for 8 weeks - Regular platelet transfusions required |
| Days 40–48 | Clinical improvement | - Stepped down to hematology ward - Ongoing chemotherapy and monitoring - Hb 89 g/L, WCC 10.6 x 10^9 /L, platelets 49 x 10^9/L on day 48 |
| Day 49 | Seizures commence | - **Continuous right-sided facial twitching** |
| Days 50–69 | Transferred to neurology ward | - **Ongoing right-sided facial twitching**, persistent fevers, clinically disorientated and dysphasic - Plain CT head: normal - CT head with contrast: enhancing nodule in right cerebellar hemisphere, suspected tuberculoma - **MRI head** with contrast: four small, contrast-enhancing, foci within the parenchyma of the brain, **likely tuberculomata**, **one in left hemisphere motor strip** corresponding with right-sided facial twitching - **EPC was diagnosed** and twitching continued until day 56**. AED dose-escalation had been required** to terminate seizures with the final regimen of phenytoin 550 mg OD, levetiracetam 1250 mg BD, and clonazepam 1 mg OD - **CSF culture: *Mycobacterium tuberculosis*** - EEG: muscle artefact |
| Days 70–124 | Transferred to infectious diseases unit for investigation of ongoing pyrexia: infection excluded as cause; pyrexia felt to be HLH-related; cyclosporine commenced | - **Fevers ongoing** - **Repeat CXRs** on day 70 and day 91 demonstrated static bilateral upper lobe fibrosis and scaring, no **new changes to explain pyrexia** - **Repeat CT-CAP** on day 93 demonstrated a mix of cavitating and calcified lung lesions and infiltrative upper zone changes in keeping with known TB, **no new features to explain pyrexia** - **Repeat MRI head with contrast** on day 94 showed increased number of enhancing parenchymal foci in keeping with tuberculomata, **no new features to explain pyrexia** - **Repeated sets of cultures including blood, urine, sputum, viral throat swabs, and blood-borne viruses remained negative** - **Repeat bone marrow aspirate** on day 114 showed **ongoing hemophagocytosis**, **ferritin had remained high** ranging 2422–4327 μg/L, and it was felt ongoing HLH could explain the pyrexia: **cyclosporine commenced** |
|  | Failed AED dose reduction as seizures recurred | - **Reduction in dose of phenytoin and clonazepam attempted.** On day 91, patient was off clonazepam and down to 350 mg daily of phenytoin when **right-sided facial twitching recurred** - Transferred to neurology ward - Seizures continued for 72 hours, had required an increased phenytoin dose to 400 mg OD, increased levetiracetam dose to 1500 mg BD, and introduction of clobazam 10 mg BD to terminate - Transferred back to the infectious diseases unit seizure-free |
| Days 125–168 | Clinical improvement, ongoing rehabilitation, discharged from hospital | - **Fevers settled, remained seizure-free** - Hb 117 g/L, WCC 7.2 x 10^9 /L, PLT 248 x 10^9/L, ferritin 860 ug/L - No material change in MRI brain appearances from before (multiple tuberculomata scattered throughout the brain) - Memory and speech improving - Ongoing physiotherapy, occupational therapy, and speech-and-language therapy - Normal immunology screen for familial HLH (granule release assay and perforin expression) - **Discharge medications included anti-TB therapy and prednisolone, levetiracetam 1500 mg, phenytoin 350 mg OD, clobazam 10 mg BD, and cyclosporine 250 mg BD** - Discharged with community rehabilitation arranged and outpatient neurology, hematology and infectious diseases follow-up |
| Abbreviations: *Hb* Hemoglobin, *g* Grams, *L* liter, *WCC* White cell count, *PLT* Platelets, *μg* Microgram, *CRP* C-reactive protein, *CXR* Chest x-ray, *HLH* Hemophagocytic lymphohistiocytosis, *HIV* Human immunodeficiency virus, *ITU* Intensive care unit, *PCP* Pneumocystis pneumonia, *CPAP* Continuous positive airway pressure, *CT-CAP* Computed tomography of the chest, abdomen, pelvis, *IV* intravenous, *TB* tuberculosis, *PCR* Polymerase chain reaction, *EPC* Epilepsia partialis continua, *CSF* cerebrospinal fluid, *AED* antiepileptic drug, *EEG* Electroencephalogram. *MRI* Magnetic resonance image, *mg* Milligram, *OD* once daily, *BD* twice daily | | |
