# Supplementary material for: Epilepsia partialis continua complicated by disseminated tuberculosis and hemophagocytic lymphohistiocytosis: a case report
Source: J Med Case Rep. 2019 Jun 24;13:191. doi: 10.1186/s13256-019-2092-x (PMC6589876; doi:10.1186/s13256-019-2092-x)
Supplement: Supplementary file 3 — Table S2. Summary of published studies reporting the co-occurrence of epilepsia partialis continua (EPC) and tuberculosis (TB). A case series summarized from the published studies reporting the co-occurrence of epilepsia partialis continua (EPC) and tuberculosis (TB). (DOCX 21 kb) [file 13256_2019_2092_MOESM3_ESM.docx]

**Table S2: Summary of published studies reporting the co-occurrence of epilepsia partialis continua and tuberculosis**

| Study | Country | Summary |
| --- | --- | --- |
| Bataduwaarachchi 2015 [1] | Sri Lanka | A 17-year-old boy on long-term immunosuppressants, for nephrotic syndrome, developed EPC. *Mycobacterium tuberculosis* was detected in his CSF. MRI of the brain demonstrated progressive vasculitic infarctions of the cerebral cortex and basal ganglia, thought to be resultant from the hypercoagulable state of TB. There was no evidence of autoimmune or viral disease. |
| Sinha 2007 [2] | India | Two patients with EPC were found to have an underlying etiology of CNS tuberculosis, with a CT brain confirming tuberculomas in both cases. |
| Pandian 2002 [3] | India | EPC was caused by tuberculous meningitis in two patients, and tuberculomas in two other patients. |
| Kravljanac [4] | Serbia | Three pediatric cases of EPC with underlying CNS tuberculosis. The duration of EPC ranged from hours to 10 days across these studies. |
| Abbreviations: *EPC* epilepsia partialis continua, *CSF* cerebrospinal fluid, *MRI* magnetic resonance imaging, *TB* tuberculosis, *CNS* central nervous system, *CT* computed tomography | | |

**References**

1. Bataduwaarachchi VR, Tissera N: **Seizures in an immunocompromised adolescent: a case report**. *J Med Case Rep* 2015, **9**:184.

2. Sinha S, Satishchandra P: **Epilepsia Partialis Continua over last 14 years: experience from a tertiary care center from south India**. *Epilepsy Res* 2007, **74**(1):55-59.

3. Pandian JD, Thomas SV, Santoshkumar B, Radhakrishnan K, Sarma PS, Joseph S, Kesavadas C: **Epilepsia partialis continua--a clinical and electroencephalography study**. *Seizure* 2002, **11**(7):437-441.

4. Kravljanac R, Jovic N, Djuric M, Pekmezovic T: **Immune-Mediated and Inflammatory Disease in Etiology of Epilepsia Parialis Continua in Children**. *Epilepsia* 2010, **51**:3-3.
